# Volatile organic compounds of *Metarhizium brunneum* influence the efficacy of entomopathogenic nematodes in insect control

**DOI:** 10.1016/j.biocontrol.2020.104527

**Published:** 2021-04

**Authors:** Esam H. Hummadi, Alexander Dearden, Tomas Generalovic, Benjamin Clunie, Alexandria Harrott, Yarkin Cetin, Merve Demirbek, Salim Khoja, Dan Eastwood, Ed Dudley, Selcuk Hazir, Mustapha Touray, Derya Ulug, Sebnem Hazal Gulsen, Harun Cimen, Tariq Butt

**Affiliations:** a Department of Biotechnology, College of Science, University of Diyala, Diyala, Iraq; b Department of Biosciences, College of Science, Swansea University, SA2 8PP, UK; c School of Medicine, Swansea University, Singleton Park, SA2 8PP, UK; d Department of Biology, Faculty of Arts and Sciences, Aydin Adnan Menderes University, Aydin, Turkey

**Keywords:** *Metarhizium brunneum*, Entomopathogenic nematodes, Volatile organic compounds, Nematicide, Semiochemicals

## Abstract

The entomopathogenic fungus (EPF) *Metarhizium brunneum* occupies the same ecological niche as entomopathogenic nematodes (EPN), with both competing for insects as a food source in the rhizosphere. Interactions between these biocontrol agents can be antagonistic or synergistic. To better understand these interactions, this study focussed on investigating the effect of *M. brunneum* volatile organic compounds (VOCs), 1-octen-3-ol and 3-octanone, on EPN survival and behaviour. These VOCs proved to be highly toxic to the infective juveniles (IJs) of the EPN *Steinernema carpocapsae*, *Steinernema feltiae* and *Heterorhabditis bacteriophora* with mortality being dose dependent. Chemotaxis studies of *H. bacteriophora* IJs in Pluronic F127 gel revealed significant preference for the VOCs compared with controls for all tested concentrations. The VOCs also impacted on the test insects in a dose-dependent manner with 3-octanone being more toxic to *Galleria mellonella*, *Cydia splendana* and *Curculio elephas* larvae than 1-octen-3-ol. Mortality of *C. splendana* and *G. mellonella* larvae was significantly higher when exposed to relatively high doses (>25%) of 3-octanone. Lower doses of 3-octanone and 1-octen-3-ol immobilised test insects, which recovered after exposure to fresh air for 2 hrs. In depth studies on *H. bacteriophora* showed that exposure of IJs to > 10% concentration of 3-octanone or 1-octen-3-ol negatively affected infectivity whereas exposure to lower doses (0.1%, 0.01%) had no effect. The VOCs affected IJs, reducing penetration efficacy and the number of generations inside *G. mellonella* but they failed to inhibit the bacterial symbiont, *Photorhabdus kayaii*. The ecological significance of VOCs and how they could influence EPF-EPN insect interactions is discussed.

## Highlights

• *Metarhizium* VOCs, 1-octen-3-ol and 3 octanone, influence behaviour of EPN.
• High doses of 1-octen-3-ol and 3 octanone repel or kill EPN infective juveniles.
• *Metarhizium* volatiles reduce EPN infectivity of insect hosts.
• *Metarhizium* VOCs immobilise or kill insects.
• Volatiles affect reproduction of EPN inside host.

## Graphical abstract

**Figure:**
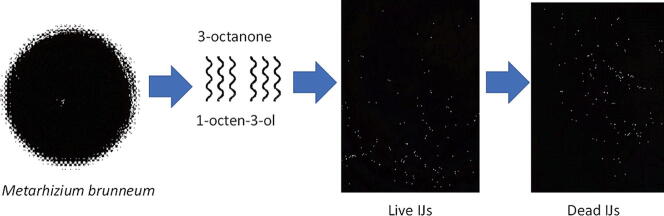

## 1 Introduction

The entomopathogenic fungi (EPF) *Metarhizium* and *Beauveria* and the entomopathogenic nematodes (EPN) *Steinernema* and *Heterorhabditis* are considered environmentally friendly alternatives to conventional chemical pesticides with several species having been developed for control of arthropod crop pests (De Faria and Wraight, 2007, Copping, 2009, Lacey et al., 2015). EPN and EPF occur naturally in the soil, occupying similar habitats and overlapping in their host range. As biological control agents (BCAs), they have complementary attributes: EPF having a better shelf life or persisting longer following application than EPN but being slower acting than EPN. EPF infect susceptible hosts through direct penetration of the cuticle using a combination of enzymes and mechanical force (Butt et al., 2016). Once inside the insect, EPF colonise the haemocoel as hyphal bodies or blastospores with the aid of proteolytic enzymes and secondary metabolites and when nutrients have been exhausted the fungus emerges to sporulate at the host surface (Butt et al., 2016). The secondary metabolites include immune modulators and antimicrobial compounds which assist in debilitation of the host and exclusion of opportunistic saprophytic microbes, respectively (Butt et al., 2016). EPN enter the host through natural orifices (mouth, anus, spiracles) but *Heterorhabditis* species can penetrate the host cuticle with the aid of a terminal “tooth” (Bedding and Molyneux, 1982, Koppenhöfer et al., 2007). Once inside the haemocoel, EPN release symbiotic bacteria which digest the host contents. EPN feed upon and multiply within the liquefied soup re-ingesting the bacteria in the process. The bacteria also protect the cadaver against other microorganisms, including EPF, through the production of a range of antimicrobial compounds (Acevedo et al., 2007, Lewis et al., 2015).

There is much evidence that pest control can be improved using combinations of EPF and EPN particularly when interactions between these BCAs are synergistic or additive (Ansari et al., 2008, Correa-Cuadros et al., 2016, Hussein et al., 2016). True synergies, where control is considerably higher than using either agent alone, allows for application rates to be significantly reduced, offering savings for end users (Ansari et al., 2008, 2010; Hussein et al., 2016). The underlying mechanism for this synergy is poorly understood but it is postulated that one agent may stress or alter the behaviour of the target insect (e.g. feeding, movement) increasing its susceptibility to the other control agent (Ansari et al., 2008). For example, EPF infected insects may be less mobile giving EPN more time to infect the host (Ansari et al., 2004). Synergy has been reported using combinations of *Metarhizium anisopliae* and *Heterorhabditis bacteriophora* against larvae of the barley chafer, *Coptognathus curtipennis* (Anbesse et al., 2008), Welsh chafer, *Hoplia philanthus* (Ansari et al., 2006) and black vine weevil (BVW), *Otiorhynchus sulcatus* (Ansari et al., 2008). Synergy was also observed using combinations of *M. anisopliae* and *Steinernema kraussei* for the control of BVW larvae at low temperatures (Ansari et al., 2010) and *Beauveria brongniartii*-*Steinernema carpocapsae* and *Beauveria bassiana-H. bacteriophora* combinations have resulted in significantly higher larval mortality of *Exomala orientalis* (Choo et al., 2002) and *Spodoptera exigua* (Barbercheck and Kaya, 1991), respectively.

Some EPF-EPN interactions are however antagonistic with the antagonism being attributed to one or more factors such as the strain of EPN or EPF, the order or timing of application, and metabolites produced by EPF or EPN symbiotic bacteria during the infection process (Shapiro-Ilan et al., 2004, Tarasco et al., 2011, Batalla-Carrera et al., 2013, Hussein et al., 2016). For example, *Steinernema feltiae* development is impaired inside Colorado potato beetle (*Leptinotarsa decemlineata*) if applied >24 hr after the fungal pathogen, *Isaria fumosorosea* (Hussein et al., 2016)*,* possibly due to *I*. *fumosorosea* metabolites negatively affecting both the developing nematodes and their symbiotic bacteria. A similar negative effect was observed in the interactions between *Metarhizium anisopliae* and *Steinernema glaseri* (Ansari et al., 2004) and *H*. *bacteriophora* (Acevedo et al., 2007) and between *B. bassiana* and S*teinernema ichnusa* (Tarasco et al., 2011).

In the rhizosphere, where critical EPF-EPN interactions usually take place, low molecular weight volatile organic compounds (VOCs) emitted by plant root associated bacteria and fungi are known to inhibit or kill plant parasitic nematodes (Gu et al., 2007, Freire et al., 2012). For example, the rhizobacteria *Bacillus megaterium* and *Lysinibacillus mangiferahumi,* the plant pathogen *Fusarium oxysporum* and mycoparasitic BCA *Trichoderma* sp. produce nematicidal VOCs active against the root-knot nematode *Meloidogyne incognita* (Huang et al., 2010, Freire et al., 2012, Yang et al., 2012a, Yang et al., 2012b). Nematicidal VOCs are produced by other non-EPF fungi including the endophytes *Daldinia concentrica* and *Muscodor albus*, and the yeast *Saccharomyces cerevisiae* with activity demonstrated against both free living (*Panagrellus redivivus*) and plant parasitic (*Bursaphelenchus xylophilus*, *M. incognita, M. javanica*) nematodes (Grimme et al., 2007, Gu et al., 2007, Riga et al., 2008, Fialho et al., 2012, Liarzi et al., 2016). There are very few reports of microbial VOCs influencing EPN behaviour or survival. One notable study is that of Wu and Duncan (2020) where the EPN *Steinernema diaprepesi* was shown to be attracted to the volatiles 1-pentanol and 1-octen-3-ol, produced by the saprophytic fungus *Fusarium solani*. Although EPF are known to produce VOCs (Crespo et al., 2008, Mburu et al., 2011, Hussein et al., 2016) most attention has focused on their role in influencing insect behaviour, particularly their attractant and repellent properties (Butt et al., 2016). Recently, Khoja et al. (2021) showed that *Metarhizium brunneum* VOCs influenced the behaviour of the root knot nematode, *Meloidogyne hapla*, being attractive at low doses and toxic at high doses. The main aims of this study were to establish if *M. brunneum* VOCs influenced EPN survival, behaviour and efficacy in killing insects. The significance of the nematicidal VOCs to *Metarhizium* ecology and pest management is discussed.

## 2 Materials and methods

### 2*.*1 Metarhizium brunneum VOCs

Details of the origin and maintenance of *M. brunneum* strains ARSEF 4556, ARSEF 3297 and V275 are given in Ansari and Butt (2011). Headspace VOCs were collected from cultures grown on Sabouraud dextrose agar media (SDA) in Bijoux vials using a 50/30 mm Divinylbenzene /Carboxen/Polydimethylsiloxane solid phase microextraction (SPME) fibre (Supelco, Bellefonte, PA, USA). Samples were analysed using an Agilent 6890 N Gas Chromatograph equipped with an HP-5MS fused capillary column (30 m × 0.25 mm × 0.25 µm film thickness), interfaced directly with an Agilent 5975 mass spectrometer. Twelve of over 40 VOCs detected were validated using authentic samples purchased from Sigma-Aldrich. These included: 3-methyl-1-butanol, isopentyl formate (isoamyl formate), methyl isovalerate (methyl 3-methylbutanoate), 3-octanone, (r)-(+)-limonene, 3-methylbutanoic acid (isovaleric acid), 1-octene-3-ol, farnesene, 2,3-butanediol, 1-octene, undecane, and tridecane.

### 2.2 Maintenance of test EPN and insects

For the initial screening of VOCs, infective juveniles (IJs) of the EPN *Steinernema carpocapsae*, *S. feltiae* and *Heterorhabditis bacteriophora*, kindly provided by BASF Ltd (UK), were used. For behavioural and infectivity studies we used IJs of *H. bacteriophora* strain 09–20, isolated from Turkish soil (Hazir et al., 2003). The EPN were stored at 4 °C until required. Viability was ≥ 90% for the control in all the experiments. Nematodes that did not move even after prodding were considered dead (De Nardo and Grewal, 2003).

Test insects included larvae of waxmoth (*Galleria mellonella,* Lepidoptera: Pyralidae), chestnut tortrix (*Cydia splendana,* Lepidoptera: Tortricidae) and chestnut weevil (*Curculio elephas*, Coleoptera: Curculionidae). Waxmoth larvae were kept in a glass jar at 25 ± 4 °C and provided an artificial diet consisting of 22% ground wheat, 22% ground maize, 11% honey, 11% glycerol, 11% milk powder, 5.5% yeast extract and 17.5% bee wax (Cakmak et al., 2013). Larvae of the chestnut weevil and chestnut tortrix were obtained from a chestnut processing factory in Aydin, Turkey, and used within 3 days of collection (Karagoz et al., 2009).

### 2.3 Preliminary screening of 12 VOCs for nematicidal activity

Twelve authenticated *M. brunneum* VOCs (Isoamyl alcohol, Isoamyl formate, Methyl isovalerate, 3-Octanone, (R)-(+)-Limonene, Isovaleric acid, 1-Octene-3-ol, Farnesene, 2,3-Butanediol, 1-Octene, Undecane, Tridecane) were screened for nematicidal activity against *S. carpocapsae*, *S. feltiae* and *H. bacteriophora.* A of nematode suspension (750 µl) containing approximately 5000 IJs in distilled water, was spread uniformly over the water agar surface (2.6% w/v agar, 5 mM potassium phosphate pH 6, 1 mM CaCI_2_ and 1 mM MgSO_4_) and acclimatised in darkness for 3 hr at room temperature. The IJs were then exposed to 20 µl VOC dispensed on an 8 mm paper disc (Whatman™, 0.34 mm thickness) positioned on a 25 × 25 mm glass coverslip placed in the centre of the Petri dish lid (Supplementary Fig. 1A). The plates were sealed with a double layer of Parafilm™, incubated in the dark at 21 °C, and checked after 24 hr. To determine EPN viability, a transect was drawn across the centre of the dish. Three 10 mm diameter solid circles were drawn along a transect starting from the centre of the plate, which were spaced 10 mm apart (Supplementary Fig. 1B). Each zone corresponded with different concentrations of the VOC. The highest concentration of the VOC was in the centre (immediately beneath the loaded filter paper), the lowest in the outer zone and intermediate concentrations in the middle zone. The dead and live nematodes in these circles were counted using a stereo binocular microscope (30X). The nematodes were classified as alive if they were mobile and possessed a J or sigmoidal shape but were considered dead if they were straight and unable to move after being probed with a needle. Mean mortality was calculated from the total number of live and dead individuals across all circles. There were five replicates per treatment and the whole experiment was repeat twice.

### 2.4 Time and dose dependent mortality assay for the EPN

From the initial screening outlined above, two compounds, 3-octanone and 1-octen-3-ol, with superior nematicidal activity were selected for dose and speed of kill assessment. The assays were performed as described previously, except that the EPN were exposed to different doses of the VOCs (5, 10, 15 and 20 µl) and mortality recorded 3, 6, 12 and 24 hr post-treatment. Controls included EPN group only and EPN and 1-octene (weak nematicide). There were five replicates per treatment and the whole experiment was repeated twice.

### 2.5 Chemotaxis of EPN in relation to VOCs

EPN chemotactic responses to 3-octanone and 1-octen-3-ol were evaluated using the modified chemotaxis assays of O’halloran and Burnell, 2003, Wang et al., 2009 with Pluronic F-127 used as substrate. A 28.75% (w/v) Aqueous Pluronic F-127 gel medium (2.5 ml) was poured on one side of a microscope glass slide (25 mm × 75 mm) and three equal zones (Z1, Z2 and Z3) marked on the agar free side. A 30 µl suspension of 100 IJs in distilled water was placed in the centre of the slide (Z2) and 1 µl distilled water on the left (Z1) and a 1 µl of VOC placed on the right side (Z3) (Supplementary Fig. 2). VOCs were tested at three different concentrations (0.01%, 1% and 100%, v/v) prepared in ethanol (99%) and used immediately in the bioassay. Each slide was placed in a Petri dish and incubated in the dark at 23 ± 2 °C. Infective juveniles were counted in each zone after 3, 6 and 24 hr. Sterile distilled water with dissolved ethanol was used as control in Z3. There were five replicates per treatment and the test was repeated three times.

### 2.6 Effects of VOCs on insects

The assays were conducted in 50 ml Falcon tubes using sterilized loamy-clay soil with 18.8% (v/w) moisture content. First, a 50 mm long X 7.13 mm wide Sharrow cellulose filter tip (Wilsons & Co. Ltd) impregnated with 100 µl VOC was placed on top of 5 g of soil at the bottom of the tube then covered with 13 g soil. Two test insect larvae (*C. elephas, C. splendana* and *G. mellonella*) per treatment were placed on the soil surface and covered with 10 g of soil (Supplementary Fig. 3A). Different dilutions of 3-octanone and 1-octen-3-ol (100, 50, 25, 12.5, 6.25 and 3.125%) were tested. Serial dilutions of VOC were prepared in 99% DMSO (Merck, Germany). Insects in the control group were exposed to filter tips infused with DMSO only. The tubes were incubated at 23–24 °C in the dark and after 48 hr the number of dead insects were recorded. The insects were exposed to fresh air for 24 hr and those that recovered were considered to have been immobilised by the VOC but those insects that failed to recover were considered dead. There were five replicates per treatment and the whole study repeated three times.

### 2.7 Effect of VOCs on EPN killing efficacy

To determine if 3-octanone and 1-octen-3-ol interfered with *H. bacteriophora* IJ infection of test insects, assays were conducted using different concentrations (100, 10, 1, 0.1 and 0.01% dissolved in DMSO) of the VOCs, dispensed from Sharrow cellulose filters (50 mm long × 7.13 mm wide). Test insects included *G. mellonella* larvae exposed to IJs and VOCs and controls consisting of larvae exposed to either IJs or VOC. Briefly, the VOC (100ul)-treated cellulose filter was placed on top of 5 g of soil at the bottom of a Falcon tube then covered with 13 g soil. The IJs (200 suspended in 100 µl distilled water) were added to each tube immediately after the addition of VOCs (Supplementary Fig. 3B) and the tubes left open for 24 hr (as would be the case if soil was fumigated with the VOCs) before placing two last instar *G. mellonella* on the soil surface. These were covered with 10 g of soil and the tubes closed with lids to prevent the insects from escaping (Supplementary Fig. 3B). For controls, IJs and/or VOCs were excluded from the treatments. The tubes were incubated at 23 ± 2 °C in the dark and larval mortality recorded 48 hr post treatment. Dead insects were incubated for one more day at room temperature before dissection under a stereomicroscope to verify nematode infection. There were five replicates per treatment and the study repeated three times.

### 2.8 Effect of VOCs on EPN penetration and reproduction inside host

Cadavers collected from the above study were incubated at 23 ± 2 °C for one more day and dissected and digested in pepsin solution (Mauleon et al., 1993). The number of IJs that penetrated each cadaver was counted at 50X magnification. To determine new generation IJs i.e. success of the EPN to reproduce inside the test insect, IJs of *H. bacteriophora* were collected every two days until no more were recovered using the White trap technique (White, 1927). The total number of emerged IJs was determined by counting three subsamples at 50X magnification (Gungor et al., 2006). For each VOC dilution, five cadavers were used and the study was conducted three times.

### 2.9 Effects of VOCs on symbiont bacteria of *H. bacteriophora*

To determine if 3-octanone and 1-octen-3-ol affected the growth of the *H. bacteriophora* bacterial symbiont, *Photorhabdus kayaii* strain 09–20 (Hazir et al., 2004, Machado et al., 2018), antibacterial assays were performed using the agar diffusion method (Qi et al., 2006). Briefly, *P. kayaii* was grown overnight in LB broth and a standardized suspension (0.5 McFarland) spread uniformly over a plate of Mueller Hinton Agar using a Drigalski spatula. Several 5 mm diameter wells were made in the agar using a transfer tube (agar borer). To each well 50 µl of pure and tenfold serial dilutions (10, 1, 0.1 and 0.01% v/v) of the VOCs were added whereas control wells contained 10% DMSO only. Cultures were incubated at 28 °C and zones of inhibition were checked after 48 hr.

### 2.10 Statistical analysis

All statistical analyses, unless indicated otherwise, were carried out using RStudio statistics package RStudio, Inc. version 1.0.153 (Team, R., 2016), using “MASS” and “multcomp” packages. For all mortality data, differential sensitivity between EPN species and/or insect species and differential compound potency were determined using a generalised linear model (GLM) and Tukey’s range test in post-hoc analysis. LD_50_ values were calculated using a GLM and the “dose.p” function within the “MASS” package. An ANOVA was carried out on the chemotactic responses of EPN to VOCs, with the strength of response compared at each time and concentration. Welch’s t-tests were used to compare means of mortality results in the final EPN efficacy assay. Data on the effects of VOCs on EPN killing efficacy were analyzed using one-way ANOVA followed by the Tukey’s test at P > 0.05 (SPSS, 2011). The mortality was adjusted for control mortality using Abbott’s formula (Abbott, 1925). Percentage data were arcsine transformed before statistical analysis.

In chemotaxis studies, differences in the number of *H. bacteriophora* IJs responding to VOCs were tested using a replicated G-test for goodness of fit (Sokal and Rohlf, 1995). The GH value tests for heterogeneity among replicates. The GP value tests for whether the pooled data deviated from a theoretical value of chance (50:50). The GT value represents sum of G values for heterogeneity (GH) and pooled data (GT). The G values are compared to a Chi-square value in tests of significance. Data on the effects of VOCs on EPN killing efficacy, penetration efficiency and generation of IJs were analyzed using one-way ANOVA followed by the Tukey’s test at P > 0.05 (SPSS, 2011). The mortality was adjusted for control mortality using Abbott’s formula (Abbott, 1925). Percentage data were arcsine transformed before statistical analysis.

## 3 Results

### 3.1 Preliminary screening of 12 VOCs for nematicidal activity

Of the 12 compounds tested, 3-octanone and 1-octen-3-ol were highly toxic, causing 85–100% mortality (Supplementary Table 1). 3-octanone caused 100% mortality in all three EPN species while 1-octen-3-ol caused 100%, 91.6 ± 2.1% and 85 ± 4% mortality of *S. feltiae, H. bacteriophora* and *S. carpocapsae,* respectively (Supplementary Table 1). The other test compounds caused mortality ranging between 0% and 58%. Most striking was the differential sensitivity of the EPN toward these compounds. In general, *S. feltiae* was highly sensitive followed by *H. bacteriophora* then *S. carpocapsae* (Supplementary Table 1). Only methyl isovalerate was more toxic to *H. bacteriophora* than *S. feltiae* causing 55 ± 2.6% *vs* 38.1 ± 7% mortality, respectively (Supplementary Table 1). 1-octene had equal weak toxicity for all three EPN species. Control mortality was 7.9 ± 0.9%, 5.4 ± 0.7% and 7.5 ± 0.9% for *H. bacteriophora, S. carpocapsae* and *S. feltiae*, respectively (Supplementary Table 1).

### 3.2 Time and dose dependent mortality assay for the EPN

**Fig. 1:**
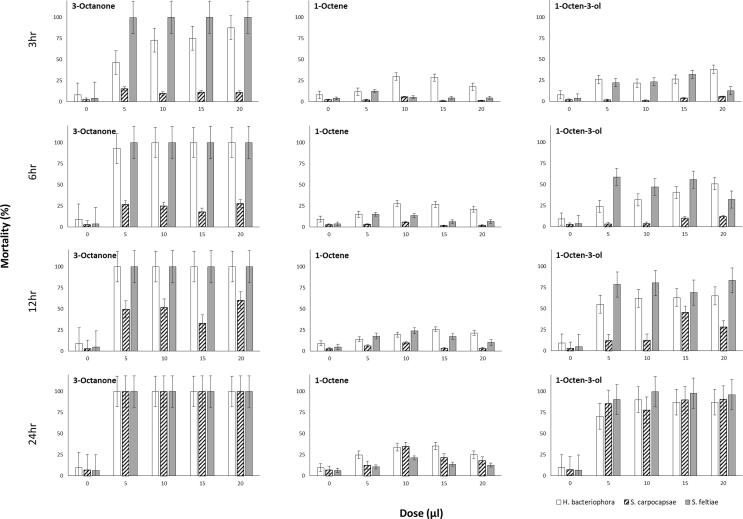
Three, 6, 12 and 24 h post exposure percentage mortality of the EPN *S. carpocapsae, S. feltiae, H. bacteriophora* exposed to *M. brunneum* VOCs at the doses 5, 10, 15 and 20 µl (0 = control group). Boxes denote interquartile range, bisected horizontally by median values; whiskers extend to 1.5 × interquartile range beyond boxes; outliers are marked as dots beyond whiskers.

The three EPN species differed in their susceptibility to 1-octen-3-ol and 3-octanone with *S. feltiae* being the most susceptible followed by *H. bacteriophora* then *S. carpocapsae* (Fig. 1). Mortality appeared to be dose-dependent for *H. bacteriophora* but less so for *Steinernema* species (Fig. 1). 3-octanone caused 100% mortality of *S. feltiae* at all doses but for *H. bacteriophora* mortality increased with dose 3 hr post treatment (Fig. 1). Interestingly, 100% mortality of *S. carpocapsae* using this compound was not observed until 24 hr post treatment at which point all doses proved fatal (Fig. 1). 1-octen-3-ol was slightly less toxic with earliest mortality recorded for *S. feltiae* and *H. bacteriophora* 3 hr post treatment and increased with time (Fig. 1). Comparatively lower mortality was recorded for *S. carpocapsae* exposed to 1-octen-3-ol after 3 hr even at the higher doses (Fig. 1). Although mortality increased with time it never reached 100% even at highest dose after 24 hr post treatment (Fig. 1). 1-octene was confirmed as the least toxic of the three VOCs tested independent of dose and time (Fig. 1). IJs of *H. bacteriophora* appeared to be more sensitive to this compound than IJs of *Steinernema* species (Fig. 1).

Mortality was higher in the centre of the Petri dish and decreased with the distance from the centre, corresponding with VOC exposure rate. Interestingly, 1-octen-3-ol was observed induce a physical response in the outer perimeter of the arena whereby IJs wriggled vigorously compared to the control groups and other compounds. For each species, an LD_50_ value was calculated at the most appropriate time point. LD_50_ values could not be obtained for EPN exposed to 1-octene because the mortality was too low or for *S. feltiae* exposed to 3-octanone because mortality was > 99% at all time points (Table 1). The LD_50_ of 3-octanone against *H. bacteriophora* was 5.68 µl ± 1.03 after 3 hr but could not be calculated at later time points because the mortality exceeded 90%. The LD_50_ of *H. bacteriophora* exposed to 1-octen-3-ol was 21.72 µl ± 1.05 after 6 hr (Table 1). For *S. carpocapsae,* the LD_50_ values of 3-octanone and 1-octen-3-ol were 94.88 µl ± 8.27 after 12 hr and 41.01 µl ± 1.06 after 24 hr, respectively (Table 1). The LD_50_ values could not be calculated for *S. carpocapsae* exposed to 3-octanone after 24 hr because all nematodes were dead at all doses (Table 1). For 1-octene, both *Steinernema* species showed low mortality with no statistical difference (*Est. =* -0.33, *p* < 0.721). However, after 24 hr, the mortality was slightly higher for both species, but did not exceed 50% with no significant difference was found between EPN species (Table 1). *S. carpocapsae* was much more tolerant of 1-octen-3-ol than *S. feltiae* (*Est. =* -3.00, *p* < 0.001) and *H. bacteriophora* (*Est. =* 3.13, *p* < 0.001) at median dose of 10 µl. Conversely*,* there was no significant difference in mortality between *S. feltiae* and *H. bacteriophora* (*Est. =* − 0.12, *s.e.* = 0.24, *z* = 0.51, *p* < 0.866) (Fig. 1).

**Table 1:** LD_50_ (±SE) values for the EPN, *S. carpocapsae, S. feltiae, H. bacteriophora,* exposed to highly toxic (1-octen-3-ol, 3-octanone) and a low toxicity (1-octene) VOCs of *M. brunneum*. The LD_50_ values were determined at specific periods post-treatment, where a= >99% mortality at 3 hr and b = mortality too low across all time periods to calculate an accurate LD_50_, therefore no nematicidal effect was assumed.

Species	Compound	Time (hr)	LD_50_ (µl)	(±SE)
*S. carpocapsae*	1-octen-3-ol	24	41.01	1.06
*S. feltiae*		6	10.56	1.04
*H. bacteriophora*		6	21.72	1.05
*S. carpocapsae*	3-octanone	12	94.88	8.27
*S. feltiae*		*a*	*a*	*a*
*H. bacteriophora*		3	5.68	1.03
*S. carpocapsae*	1-octene	*b*	*b*	*b*
*S. feltiae*		*b*	*b*	*b*
*H. bacteriophora*		*b*	*b*	*b*

1-octene was the least toxic compound with *H. bacteriophora* demonstrating greater sensitivity to this compound than *S. feltiae* at 10 µl (*Est*. = 2.27, *s.e.* = 0.34, *z* = 6.75, *p* < 0.001) and also *S. carpocapsae* at the same dose (*Est.* = 2.60, *p* < 0.001).

### 3.3 Chemotaxis response of *H. Bacteriophora* IJs to VOCs

**Fig. 2:**
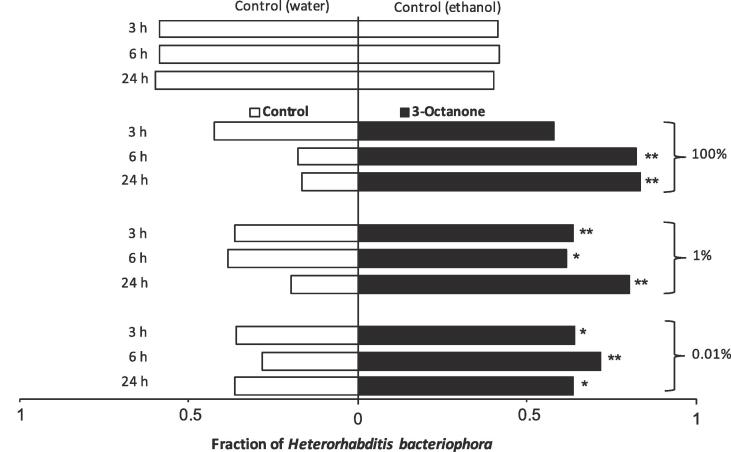
The chemotactic response of *Heterorhabditis bacteriophora* infective juveniles after 3, 6, and 24 hr when offered a choice between 3-octanone (right closed bars) and no odor source (left open bars). Each bar represents the choice of nematodes. *P < 0.05, **P < 0.01.

When offered a choice between no odour source and 3-octanone, *H. bacteriophora* IJs showed a general significant preference for VOC source compared to no odour source for all tested concentrations (Fig. 2). At 100% concentration of 3-octanone, no significant preference of IJs was observed at 3 hr assessment but 83.3% and 82.1% of the IJs were found in the side of the slide with VOC after 6 and 24 hr, respectively (Fig. 2). Overall, 63.6%, 61.4% and 80.2% of the IJs significantly preferred the side of the slides with 1% of 3-octanone after 3, 6 and 24 hr, respectively. At 0.01% concentration, statistically more (average 66.3%) of *H. bacteriophora* IJs moved towards 3-octanone volatile at each point of assessment during the experiment. In the control group, however, *H. bacteriophora* IJs did not show any preference when no odour source was used (Fig. 2).

**Fig. 3:**
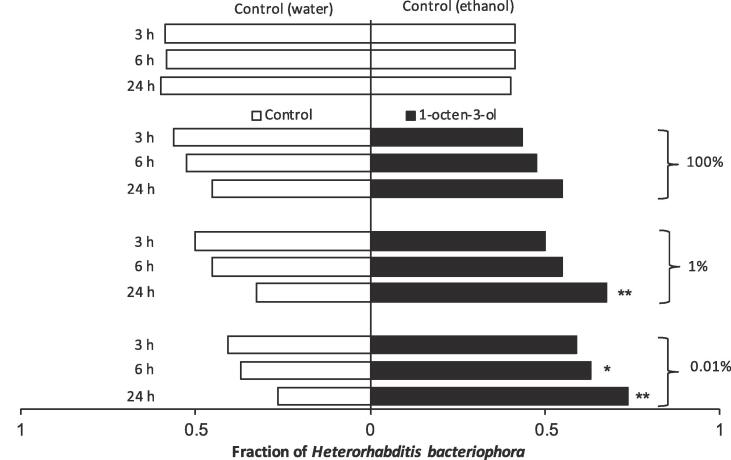
The chemotactic response of *Heterorhabditis bacteriophora* infective juveniles after 3, 6, and 24 hr when offered a choice between 1-octen-3-ol (right closed bars) and no odor source (left open bars). Each bar represents the choice of nematodes. * = *P* < 0.05, ** = *P* < 0.01.

In the case of 1-octen-3-ol, nematodes did not show any significant preference between the 100% volatile and control sides at all assessment time points (Fig. 3). At 1% VOC concentration, statistical difference was only observed after 24 hr when 67.5% of the IJ preferred the volatile side. At the lowest concentration (0.01%) of 1-octen-3-ol, significantly more *H. bacteriophora* IJs were found on the side with the volatile at 6- and 24-hours assessment periods (Fig. 3).

### 3.4 Effects of VOCs on insects

**Fig. 4:**
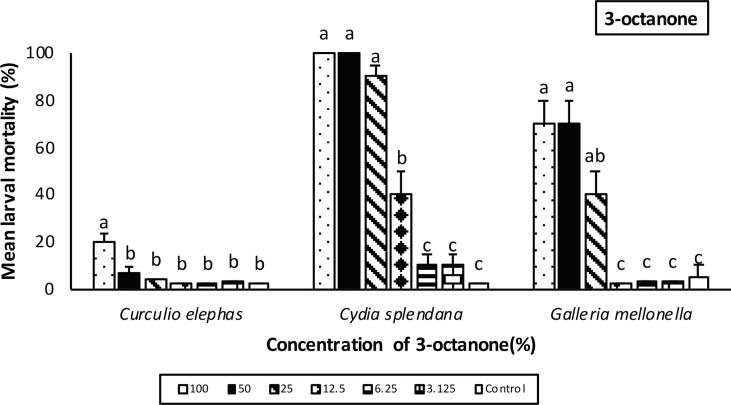
Mean percentage mortality of insect larvae (*Curculio elephas, Cydia splendana, Galleria mellonella*) exposed to 3-octanone after 48 hr. Error bars represent standard error. Lower case letters above error bars represent Tukey’s homogenous subset groups, indicating statistcal similarities and differences (*P* < 0.05; Tukey’s test).

Mortality was affected by the volatile type and concentration and insect species used. Among the insects tested *C. splendana* was the most sensitive to 3-octanone, where larval mortality was 100% for 100 and 50% concentrations of 3-octanone. At 25% concentration, larval mortality was 90 ± 5.3% but mortality decreased sharply at lower (12,5 and 6.25%) VOC concentrations. There was a significant difference between 100, 50 and 25% group and the other concentrations (F = 77.29; df = 6,98; P < 0.001) (Fig. 4). *G. mellonella* larvae was also relatively susceptible to 3-octanone with 70% (±10.6) mortality recorded for 100, 50% concentrations and 40 ± 10.2% mortality recorded for 25% volatile concentration. Significant difference was observed between these treatments and the other dilutions (F = 16.03; df = 6,98; P < 0.001) (Fig. 4). In the case of *C. elephas* larvae, only 100% concentration of 3-octanone caused significant mortality (19.8 ± 4.3%) than control (F = 12.60; df = 6,98; P < 0.001) (Fig. 4).

**Fig. 5:**
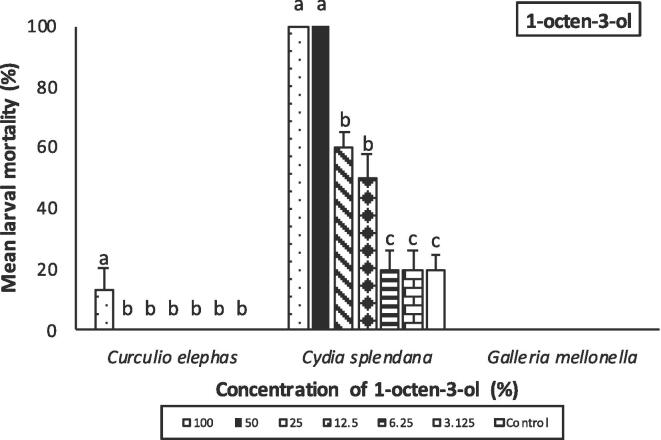
Percentage mortality of insect larvae (*Curculio elephas, Cydia splendana, Galleria mellonella)* exposed to 1-octen-3-ol after 48 hr. Data are expressed as mean ± SEM. The same lower case letter above the error bars indicates no significant difference (*P* < 0.05; Tukey’s test).

When 1-octen-3-ol was applied, 100% mortality occurred for *C. splendana* larvae exposed to 100 and 50% volatile concentration. This was the highest mortality followed by the concentrations of 25 and 12.5% which caused 60 ± 5.3% and 50 ± 8.4% mortality, respectively (F = 46.20; df = 6,98; P < 0.001) (Fig. 5). Except for *C. elephas* larvae treated with 100% 1-octen-3-ol (F = 3.50; df = 6,98; P < 0.01)*,* no larval mortality was observed in *G. mellonella* and *C. elephas* larvae for all tested concentrations (Fig. 5).

### 3.5 Effects of VOCs on EPN killing efficacy

**Fig. 6:**
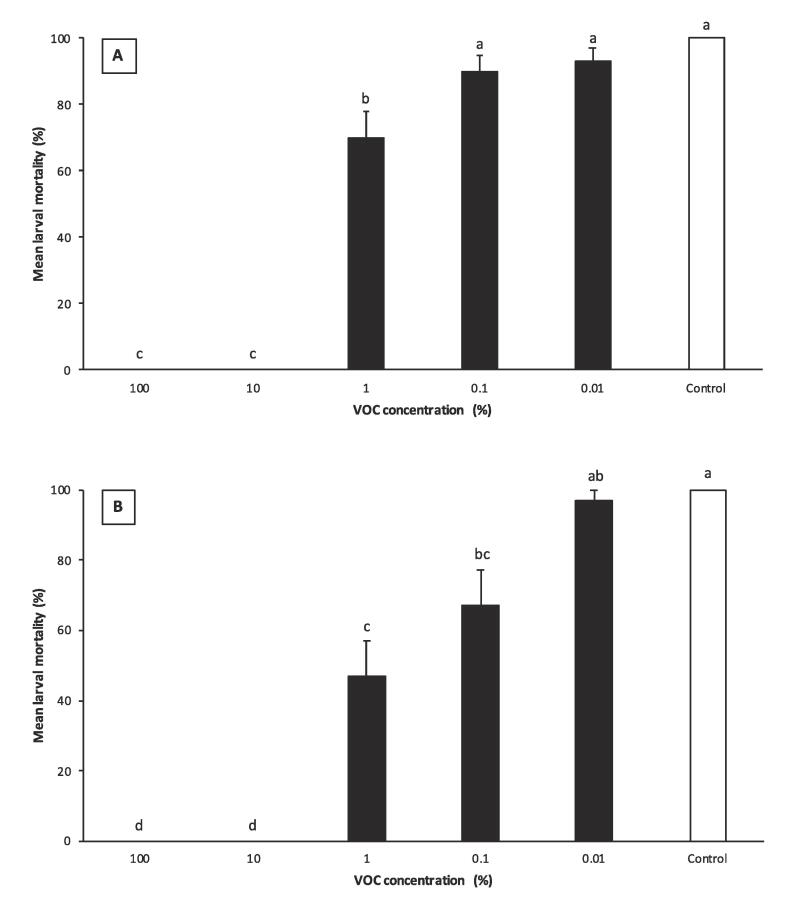
Corrected percentage mortality of *Galleria mellonella* larvae caused by nematodes exposed to 3-octanone (A) or 1-octen-3-ol (B). Data are expressed as mean ± SEM. The same lower case letter above the error bars indicates no significant difference (*P* < 0.05; Tukey’s test).

Exposure of *G. mellonella* larvae to 100% and 10%, 3-octanone resulted in 83 ± 7.9% and 13 ± 3.3% mortality, respectively. However, the same mortality was obtained in the tubes with the same concentrations of VOC used in control group and the absence of IJs in dissected cadavers exposed to IJs and 3-octanone confirmed that the death was due to the volatile, not the EPN. After the mortality correction using Abbott, the graph shows no mortality for 100 and 10% concentrations (Fig. 6). Insect cadaver in treatments with lower of VOCs (1, 0.1 and 0.01%) had IJs inside after dissection with pepsin solution and these cadavers had red color which is a unique sign of *H. bacteriophora* infection.

Exposure of *G. mellonella* larvae to *H. bacteriophora* IJs at lower doses (1, 0.1 and 0.01%) of 3-octanone resulted 70 ± 8.1%, 90 ± 6.2%and 93 ± 4.5% mortality, respectively. In the positive control, EPN killed all insect larvae. Mortality was not significantly different between the untreated control, 0.1 and 0.01% of 3-octanone treatments. However, EPN caused statistically more larval mortality than 1% dilution of 3-octanone (F = 68.94; df = 5,84; P < 0.0001) (Fig. 6A).

EPN efficacy in killing *G. mellonella* larvae increased with decreasing dose of 1-octen-3-ol, thus 1, 0.1, and 0.01% concentrations of 1-octen-3-ol resulted in 49, 68 and 97% mortality, respectively. EPN killing efficacy in the absence of VOC was 100%, which was similar to the 0.01% dose but statistically higher than the infectivity of IJs treated with 1 and 0.1% of VOC (F = 36.09; df = 5,84; P < 0.0001) (Fig. 6B).

### 3.6 Effect of VOCs on EPN penetration and the number of new generation infective juveniles

**Fig. 7:**
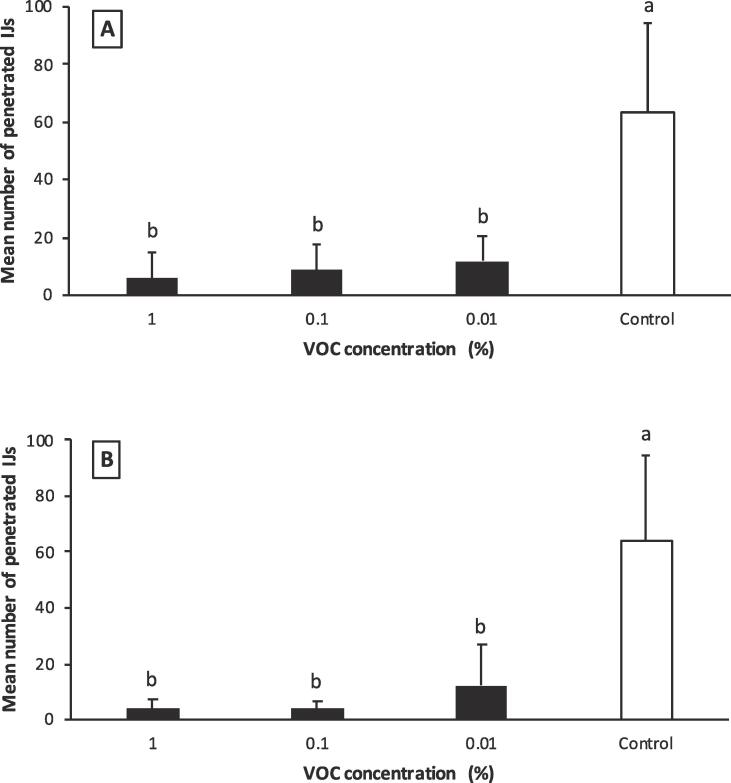
Number of penetrated nematodes per *Galleria mellonella* larva. (A) 3-octanone, (B) 1-octen-3-ol. The same lowercase letter above the bars indicates no significant difference (*P* > 0.05; Tukey’s test).

Few IJs (<15) of *H. bacteriophora* penetrated *G. mellonella* larvae in the presence of VOCs but significantly more (average 63 ± 9.8) infected in the control group. The penetration efficiency was statistically different between control and 3-octanone (F = 31.09; df = 3,36; *P* = 0.0001) (Fig. 7A) and 1-octen-3-ol (F = 27.58; df = 3,36; *P* = 0.0001) treatments (Fig. 7B). Although, the number of penetrated nematodes increase as the VOC dilution decreased, there was no statistical difference among the dilutions.

**Fig. 8:**
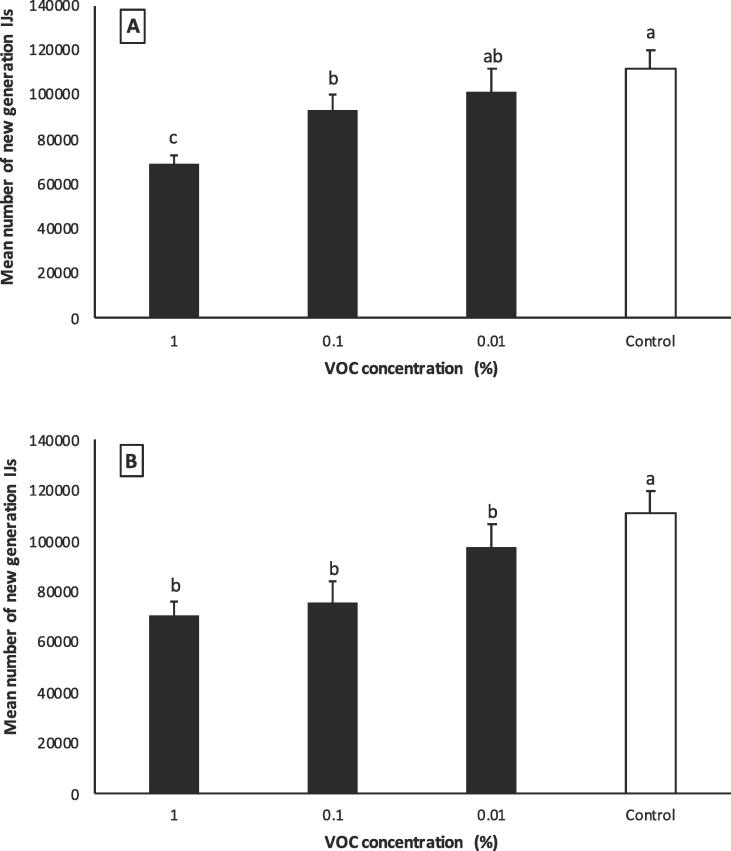
Total number of emerged IJs per *Galleria mellonella* larva. (A) 3-octanone, (B)- 1-octen-3-ol. The same lowercase letter above the bars indicates no significant difference (*P* > 0.05; Tukey’s test).

The highest number of emerged IJs was observed for the control group (average 110,000 ± 10530), and the lowest number was observed for 1% of 3-octanone (average 69,000 ± 3823). There was a significant difference between the control and 1 and 0.1% 3-octanone dilutions (F = 28.72; df = 3,36; *P* = 0.0001) (Fig. 8A). The number of emerged IJs in the control group was significantly higher than all dilutions of 1-octen-3-ol (F = 22.96; df = 3,36; *P* = 0.0001) (Fig. 8B).

### 3.7 Effects of VOCs on symbiont bacteria of *Heterorhabditis bacteriophora*

No zones of inhibition were observed in the untreated controls and VOC (3-octanone, 1-octen-3-ol) treated bacteria irrespective of dose.

## 4 Discussion

This study shows that VOCs produced by EPF affect the behaviour and survival of EPN. Of the *M. brunneum* VOCs screened the most toxic was 3-octanone followed by 1-octen-3-ol with other compounds exhibiting weak or no activity. *In vitro* studies showed that at relatively high concentrations these compounds killed EPN, independent of species, within 24hr. However, at lower concentrations they appeared to have nematistatic (immobilising) or attractant properties. Similarly, tests insects were killed at high doses but temporarily anaesthetized at relatively low doses. Most studies to date have focussed on the impact of microbial VOCs on plant parasitic and other economically important nematode species with little attention being given to EPN (Gu et al., 2007, Yang et al., 2012b, Cheng et al., 2017, Pimenta et al., 2017). It is possible that *M. brunneum* produces nematicidal compounds to repel fungivorous invertebrates which include nematodes such as *Aphelenchus* and *Aphelenchoides* species (Haraguchi and Yoshiga, 2020).

Both 1-octen-3-ol and 3-octanone are common, widespread compounds. They are produced by fungi and many plant species, forming volatile fractions of essential plant oils (Lee et al., 2005, Combet et al., 2006, Socaci et al., 2009, Xiao et al., 2017). In spite of their widespread occurrence, relatively little is known about their ecological role (Werner et al., 2016). They do constitute a tiny fraction of a vast number of diverse VOCs produced by *Metarhizium* and other soil fungi (Werner et al., 2016, Bojke et al., 2018). 1-octen-3-ol is known to attract or kill insects and inhibit germination of fungal spores (Hall et al., 1984, Takken and Kline, 1989, Chitarra et al., 2004, Zhao et al., 2011). 3-octanone attracts insects such as the phorid fly, *Megaselia halterata* and helps earthworm locate microbial food sources (Pfeil and Mumma, 1993, Zirbes et al., 2011, Davis et al., 2013). Recently, 1-octen-3-ol and 3-octanone were shown to have mollusc repellent and molluscicide properties (Khoja et al., 2019) and nematicidal effects against plant parasitic nematodes (Khoja et al., 2021).

The biological activity of 3-octanone and 1-octen-3-ol, like that of many other pesticidal VOCs, is dose-related (Khoja et al., 2019). The fact that 3-octanone was more toxic suggest its mode of action differed from that of 1-octen-3-ol. EPN were significantly more sensitive to these VOCs than the much larger insects and would be the first to be killed if the VOCs were developed as fumigants to eradicate subterranean insect pests. At moderate concentrations, the VOCs anaesthetise insects independent of species. Theoretically, these immobilised insects would be easier to infect by EPN but this study shows EPN infectivity is greatly reduced. The fact that 1-octen-3-ol and 3-octanone along with other fungal volatiles act as semiochemicals, attracting some insects and repelling others (Butt et al., 2016, Holighaus and Rohlfs, 2019). It is possible that the attractant compounds may form part of *M. brunneum* EPF “lure and kill” and/or dispersal strategy.

Chemotaxis studies showed that at relatively low concentrations 3-octanone and 1-octen-3-ol act as EPN attractants. Both volatiles from *M. brunneum* were recently shown to be attractive at low concentrations to the plant parasitic nematode, *M. hapla* (Khoja et al., 2021). The latter produced by *Fusarium solani* was shown to attract *Steinernema diaprepesi* (Wu and Duncan, 2020). Even though plants also produce these compounds they are not included in the repertoire of EPN attractant volatiles emitted by herbivore damaged roots (Jagodič et al., 2019). It is possible that EPF and plant VOCs act synergistically in luring EPN to the rhizosphere. This could benefit EPF like *M. brunneum* in several ways. For example, EPN could stress the insect host and increase its susceptibility to EPF infection and/or the EPN could increase insect movement and acquisition of conidia leading to infection (Ansari et al., 2008, Ansari et al., 2010). Interestingly, the EPN *S. diaprepsi* is attracted to conidia and mycelium of the plant pathogenic fungus *F. solani* irrespective of the presence or absence of the insect host, *Diaprepes abbreviatus,* responding to the VOCs 1-octen-3-ol and 1-pentanol (Wu et al., 2018, Wu and Duncan, 2020). *Fusarium* species are also known to produce 3-octanone (Takeuchi et al., 2012). Wu et al., (2018) further showed that the two agents worked synergistically when applied together. Although the fungus did not reduce the number of IJs entering the insect host and enhance the insecticidal efficiency of *S. diaprepesi,* it was able to exploit the resources in the cadaver. The current study shows that the number of IJs entering and multiplying in the insect host is significantly reduced in the presence of the VOCs 3-octanone and 1-octen-3-ol.

The fact that interactions between EPF and EPN can range from antagonist to synergistic suggest their relationship is much more complex. It could be argued that some strains of EPF produce VOCs to lure EPN and exploit synergies whereas others produce repellent compounds to reduce competition with EPN. It is unlikely that 1-octen-3-ol and 3-octanone kill EPN in the rhizosphere since this study shows that relatively high doses are required. Clearly more studies are needed to better understand the interactions between these two biocontrol agents since synergistic interactions will not only lead to more effective deployment but could potentially reduce application rates and costs for end users.

## 5 Conclusions

*Metarhizium brunneum* emit volatiles some of which, notably 1-octen-3-ol and 3-octanone, are toxic to IJs of *S. feltiae, S. carpocapsae* and *H. bacteriophora* with the degree of mortality being dependent on the exposure dose. The VOCs influenced EPN behaviour and the ability to infect insects by reducing the number of generations inside the host compared with healthy controls. The volatiles did not affect the symbiotic bacteria since their growth was unaffected in *in vitro* assays. These findings could help in the development of improved integrated pest management strategies using BCAs and semiochemicals.

## Declaration of Competing Interest

The authors declare that they have no known competing financial interests or personal relationships that could have appeared to influence the work reported in this paper.
