# Supplementary material for: Volatile organic compounds of Metarhizium brunneum influence the efficacy of entomopathogenic nematodes in insect control
Source: Biol Control. 2021 Apr;155:104527. doi: 10.1016/j.biocontrol.2020.104527 (PMC7923176; doi:10.1016/j.biocontrol.2020.104527)

SUPPLEMENTARY TABLE

Supplementary Table 1 Screening of *M. brunneum* VOCs for nematicidal activities against three species of entomopathogenic nematodes: *Steinernema carpocapsae, Steinernema feltiae* and *Heterorhabditis bacteriophora*.

Supplementary Table 1.

**Compound (purity)**	**Mortality (%) (Mean ± SE)**		
	S. carpocapsae	***S. feltiae***	***H. bacteriophora***
Isoamyl alcohol (99%)	6 ± 1.7	20.8 ± 3	9.7±2.1
Isoamyl formate (95%)	3.7 ± 1.3	58 ± 2.2	46.6 ±1.8
Methyl isovalerate (98%)	9.8 ± 2.9	38.1± 7	55 ±2.6
3-Octanone (98%)	100 ± 0	100 ± 0	100 ± 0
(R)-(+)-Limonene (97%)	0 ± 0	16.7 ± 3.2	5.9 ± 1.9
Isovaleric acid (99%)	4.4± 4	16.1 ± 3.8	13 ± 2.8
1-Octene-3-ol (98%)	85 ± 4.8	100 ± 0	91.6 ± 2.1
Farnesene (mixture of isomers)	1.32 ± 1	43 ± 1.1	7.8 ±3.1
2,3-Butanediol (98%)	3.7 ± 2.2	12.5 ± 7.2	11.4 ± 2.3
1-Octene (98%)	18.2 ± 0.9	20.1 ± 3	19.4 ± 3.8
Undecane (99%)	1.2 ± 1.1	25.4 ± 5.6	7.5 ± 2.7
Tridecane (99%)	2.18 ± 1.3	32.3 ± 5	4.1 ± 1.6
Control (EPN only)	5.4 ± 0.7	7.5 ± 0.8	7.9 ± 0.9

SUPPLEMENTARY FIGURES

Supplementary Figure 1 Petri dish assay to determine effect of VOC dose on survival of entomopathogenic nematodes (EPN). **(A)** Cross-section of the experimental arena shows the inverted Petri dish base filled with 20 ml of water agar medium. IJs were applied uniformly over the surface. VOCs were dispensed from a filter paper disc placed centrally on a glass coverslip in the middle of the Petri dish lid. **(B)** Plan view of test arena. Observations were made of an area (solid circle) along a transect from three zones delineated by dashed circles. Each zone corresponds to different concentrations of the VOC. The highest concentration of the VOC was in the centre (immediately beneath the loaded filter paper), the lowest in the outer zone and intermediate concentrations in the middle zone.

Supplementary Figure 2. Chemotaxis assay design. One side of glass microscope slide was coated with 2.5 ml of Pluronic F-127 gel. This clear matrix facilitates visualisation of entomopathogenic nematodes (EPN). The slide consisted of three zones (Z1, Z2, Z3) each measuring 2.5 x 2.5 cm. EPN (30 µl of 100 IJs) placed at the centre had the choice of either moving to the control (Z1), consisting of either water or a mixture of water and ethanol, or to the volatile organic compound (VOC) in Z3.

Supplementary Figure 3. Design of experiments to investigate the effect of *M. brunneum* VOCs on insect survival and EPN efficacy. **(A)** Illustrates the effect of the VOCs dispensed from a filter tip on the survival of spatially separated test insects. **(B)** Illustrates the effect of the VOCs on EPN efficacy in killing test insects.

Supplementary Figure 1.

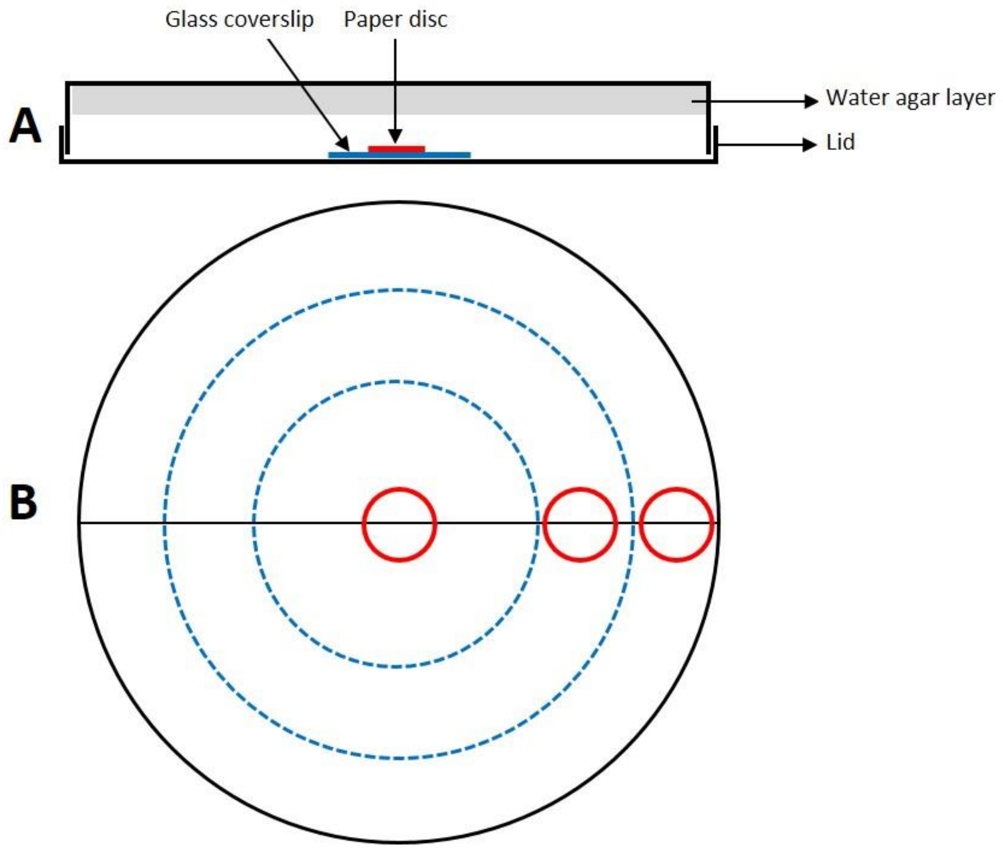

Supplementary Figure 2.

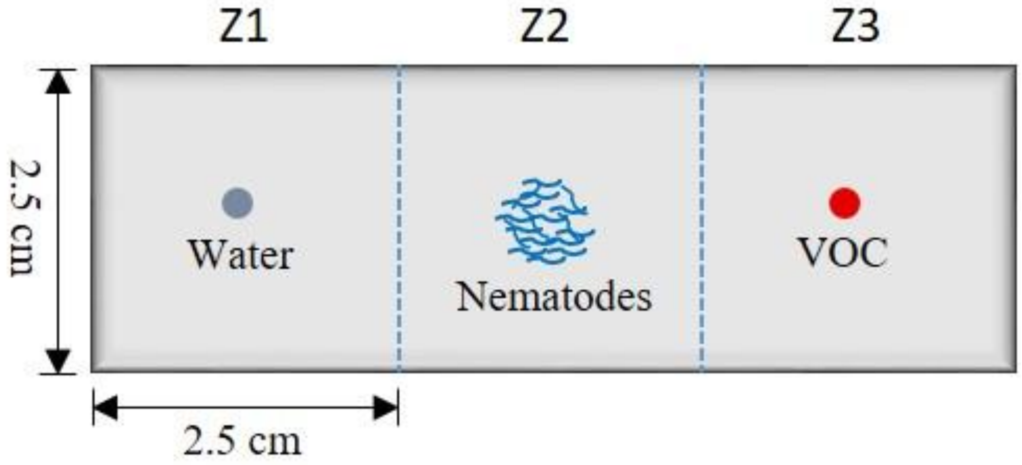

Supplementary Figure 3.

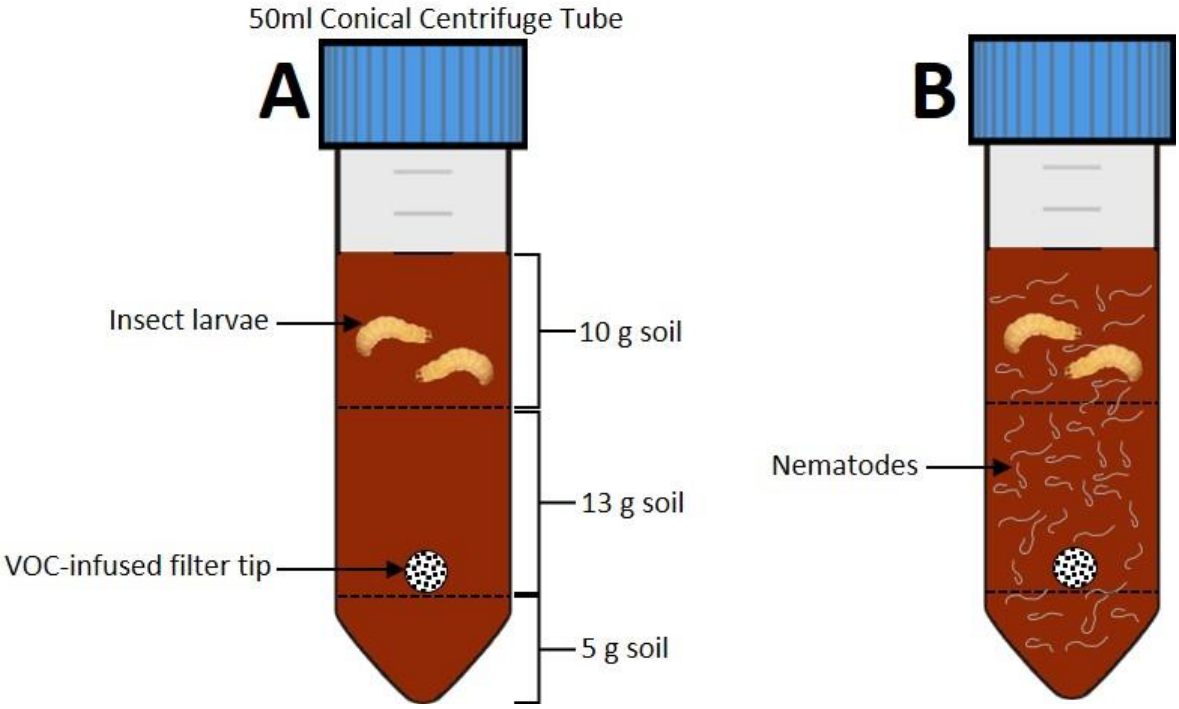

Supplement: Supplementary data 1 [file mmc1.pdf]
